# Rapidly vanishing left atrial dissection following mitral valve replacement: a case report

**DOI:** 10.1186/s13019-020-01112-3

**Published:** 2020-05-06

**Authors:** Atsushi Morishita, Seiichiro Katahira, Takeshi Hoshino, Kazuhiko Hanzawa, Hideyuki Tomioka

**Affiliations:** 1 Department of Cardiovascular Surgery, Numata Neurosurgery Heart-Disease Hospital, 8 Sakae-cho, Numata, 378-0014 Japan; 2 Division of Health Administration, Hamakawasaki Operation Center, Toshiba Human Asset Service Corporation, Kawasaki, Japan; 3 Department of Anesthesiology, Minami Machida Hospital, Machida, Japan; 4 Department of Advanced Treatment and Prevention for Vascular Disease and Embolism, Niigata University Graduate School of Medical and Dental Sciences, Niigata, Japan; 5 Department of Cardiovascular Surgery, Tokyo Women’s Medical University, Yachio Medical Center, Yachio, Japan

**Keywords:** Left atrial dissection, Mitral valve replacement, Serial transesophageal echocardiography, Conservative management, Close observation, Hemodynamics

## Abstract

**Background:**

Left atrial dissection is an extremely rare complication of mitral valve replacement. Because of its severity, its prompt diagnosis and treatment is mandatory. The most effective treatment (i.e. surgical vs. non-surgical) for left atrial dissection has not been fully established yet.

**Case presentation:**

Herein, we have reported left atrial dissection after mitral valve replacement in a 68-year-old obese woman. After closing the thorax, transesophageal echocardiography (TEE) revealed an atrial mass of 3 cm × 2 cm, visualized as an oval hypoechoic appearance extending from the posterior annulus of the mitral valve to the posterior wall of the left atrium. Because hemodynamic conditions were stable, surgery was ruled out and conservative treatment with close observation was selected. On postoperative day 2, TEE revealed that the atrial mass had vanished and the broken piece of the endocardium merely remained fluttering in the atrium. On postoperative day 6, the appearance of the left atrium was normalized completely, leaving no traces of left atrial dissection. The patient recovered uneventfully. Serial TEE was a very effective imaging modality during the non-surgical treatment of left atrial dissection.

**Conclusions:**

It is crucial to accurately define diagnosis and optimally consider therapeutic strategies for left atrial dissection based on the hemodynamic conditions of the patient and serial TEE follow-up examinations. In our case study, left atrial dissection was successfully treated with conservative treatment; therefore, we believe that TEE could be a feasible modality for the early diagnosis of this condition.

## Background

Left atrial dissection (LAD) after mitral valve replacement is an extremely rare complication [1, 2]. Its clinical presentation is very different in individual cases. Surgical treatment for LAD is often selected when the patient has unstable hemodynamic conditions. On the other hand, conservative treatments with close observation are commonly employed under stable hemodynamic conditions. Herein, we describe a case of LAD after mitral valve replacement that was treated conservatively. Transesophageal echocardiography (TEE) follow-up examinations confirmed the disappearance of LAD in its early stages.

## Case presentation

A 68-year-old woman presented to our hospital complaining of dyspnea at rest for approximately 2 months and was classified as New York Heart Association functional class III. She had previously been treated for breast and colon cancers and had not suffered from cardiovascular disease. Her height, weight, and body mass index were 150.0 cm, 91.0 kg, and 40.4 kg/m^2^, respectively. A grade IV/VI pan-systolic murmur was heard at the apex on cardiac auscultation. Her pulse rate was 105 beats/min and she was diagnosed with an irregularly rapid heartbeat (tachycardia). Her blood pressure was 124/76 mmHg. A chest radiograph revealed severe cardiomegaly with a cardiothoracic ratio of 80%, while an electrocardiogram showed persistent atrial fibrillation with a low-voltage F-wave. Computed tomography demonstrated the retention of pleural fluid or abdominal dropsy and hepatosplenomegaly. No significant coronary stenosis was observed by coronary angiography. Transthoracic echocardiography revealed severe mitral regurgitation with annular enlargement and severe tricuspid regurgitation. The cause of mitral regurgitation was severe tethering due to extreme annular enlargement, and the prolapse of the anterior leaflet. The left ventricular ejection fraction was 49%, the left ventricular diastolic diameter was 63 mm, and the left atrial diameter was 71 mm. Laboratory evaluations did not show any abnormalities, apart from an N-terminal-proB-type natriuretic peptide level of 7439 pg/ml. Because of the medication-refractory heart failure, we decided to perform surgery after a written informed consent was obtained.

**Fig. 1:**
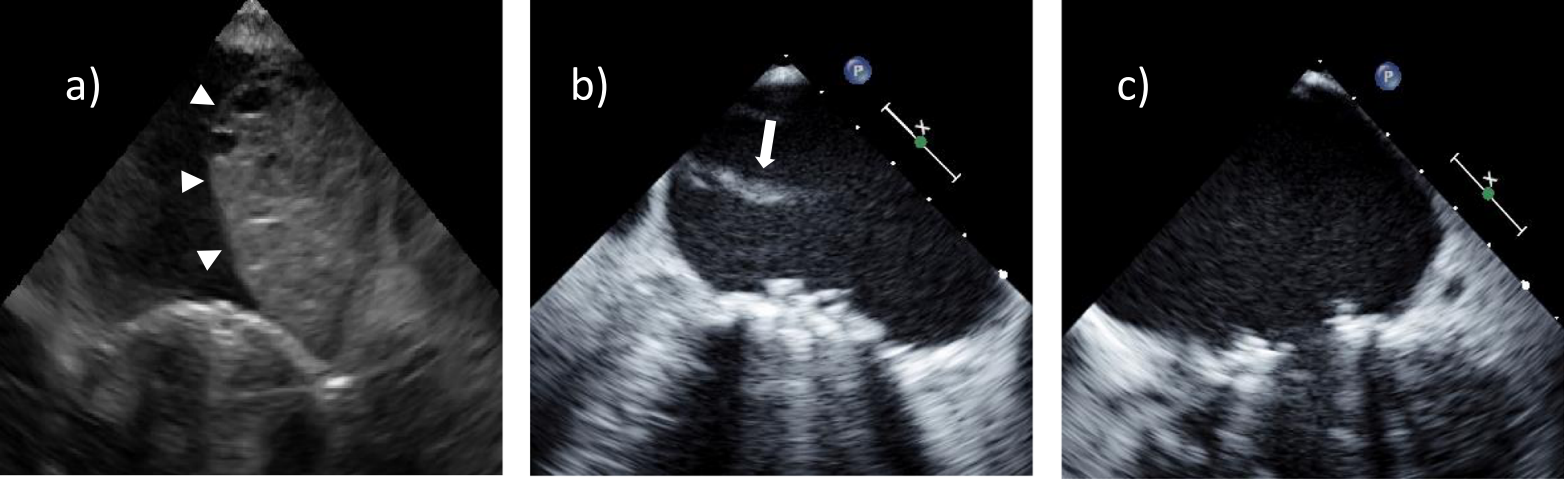
Serial transesophageal echocardiography. **a** Intraoperative transesophageal echocardiography revealing an oval hypoechoic appearance (white arrowheads) extending from the posterior annulus of the mitral valve to the posterior wall of the left atrium. **b** Transesophageal echocardiography on postoperative day 2 revealing the vanishing atrial mass and the remaining broken piece of the endocardium fluttering in the atrium (white arrow). **c** Transesophageal echocardiography on postoperative day 6 revealing the complete disappearance of the left atrial dissection

A median sternotomy was performed and standard cardio-pulmonary bypass was initiated with ascending aortic and bicaval cannulation. A left atrial vent was introduced from the right upper pulmonary vein. Myocardial protection was achieved in an antegrade and retrograde fashion. The retrograde cardioplegia cannula was inserted smoothly under direct vision. After aortic cross-clamping, the left atrium was opened through a longitudinal left atriotomy, revealing the tethering mechanism secondary to the prolapse of the anterior leaflet of the mitral valve. The clear zone of the anterior leaflet was excised and the rough zone with its attached chord were divided equally. Each divided structure was transferred to the anterolateral and the posteromedial commissures separately. The posterior leaflet, including the subvalvular apparatus, was wholly preserved. The mitral valve was replaced with a 29-mm St. Jude mechanical valve (St. Jude Medical Inc., St. Paul, MN) in an intra-annular position using everting mattress sutures. Subsequently, tricuspid annuloplasty was performed with a 32-mm Carpentier-Edwards PhysioTricuspid ring (Edwards LifeSciences, Irvine, CA) for annular enlargement. Weaning from the cardio-pulmonary bypass was successful. After closing the thorax, to our surprise, an atrial mass of 3 cm × 2 cm was detected by TEE, which showed an oval hypoechoic appearance extending from the posterior annulus of the mitral valve to the posterior wall of the left atrium (Fig. 1a). Although it occupied one-third of the left atrium, there was no significant obstruction of the pulmonary venous return and left ventricular filling. The prosthetic valve movement was unobstructed and there were no paravalvular leaks or pericardial effusion. No communication between the mass and the atrial true lumen was detected using color Doppler echocardiogram. We diagnosed the mass as LAD. Because hemodynamic conditions were stable after a consulting with the anesthesiologist, the patient was deemed unsuitable for surgery. She was transferred to the intensive care unit and remained under close observation. On postoperative day 2, TEE showed that the atrial mass had vanished and the broken piece of the endocardium merely remained fluttering in the atrium (Fig. 1b). On postoperative day 6, the appearance of the left atrium had normalized completely, leaving no traces of LAD (Fig. 1c). The patient had an uneventful postoperative course, except for prolonged respiratory failure. She was discharged from the hospital on postoperative day 47 and is currently being followed up once a month.

## Discussion and conclusions

LAD is an extremely rare complication and often occurs after mitral valve surgery. It is defined as a false blood-filled cavity that extends from the mitral annulus into the left atrial wall, with or without connection to the true left atrium [3]. Incidence rates are reported to be 0.16 and 0.84% after mitral valve surgery and replacement, respectively [1, 2]. Fukuhara et al. hypothesized that partial atrioventricular groove injury majorly contributes to LAD etiology [1]. It is important to strictly control the perioperative blood pressure in order to minimize the damage to the atrioventricular groove and prevent left ventricular rupture. A pressurized blood flow from the left ventricle across the annulus in the opposite direction of the pericardial cavity could form the dissection cavity, because of the separation of the layers of the left atrium; therefore, LAD may be classified as a subtype of type I left ventricular rupture. Extensive debridement of the posterior annulus or subvalvular apparatus (especially in the heavily calcified areas of the annulus), inappropriate suturing of or traction applied on the annulus, oversizing of the inserted prosthesis, inadvertent injury to the endocardium of the left atrium by surgical retraction, and inadequate reversal of anticoagulation may contribute to the occurrence of LAD after mitral valve surgery [4]. Furthermore, LAD is most frequently located in the posterior wall of the left atrium as reported by Fukuhara et al. [5]. In the present case, LAD occurred, even though both leaflets and their subvalvular apparatuses were completely left intact. Its apparent cause remains unknown; however, it has been speculated that valve sutures might unintentionally be placed shallowly in the posterior annulus close to the left atrium. Meanwhile, coronary artery bypass grafting, aortic valve replacement, acute myocardial infarction, blunt chest trauma, infectious endocarditis, amyloidosis, percutaneous coronary intervention, radiofrequency ablation, and spontaneous etiology have been reported as other causes of LAD [6–11]. We could not completely exclude the possibility of coronary sinus perforation by retrograde cardioplegic perfusion, although the perfusion pressure was appropriately monitored. The most common presentation time of LAD was reported to be during the procedure. The intraoperative routine use of TEE is important for early detection of atrial dissection and accurate diagnosis.

TEE findings revealed the rapid worsening in size of the false lumen (extending from the mitral annulus to the left atrial wall or atrial septum), mitral regurgitation with periprosthetic leak or malfunction, and progressive obstruction of the mitral valve inflow or pulmonary vein orifice, which indicate the low-output syndrome. Furthermore, it is crucial to determine the best time for surgery based on serial TEE follow-up examinations and the hemodynamic conditions of the patient.

Regarding therapeutic strategies for LAD, we have two available options: surgical treatment and conservative management with close observation. Surgery is often selected in order to prevent life-threatening complications; this often compromises the hemodynamics of the patient. Surgery constitutes evacuation of the hematoma, obliteration of the dissected cavity, and closure of entry and reentry [12]. When the entry to the false lumen could not be identified, marsupialization was used for releasing the pressure of the false lumen toward the right atrium [13]. In a very critical situation, it might be necessary to perform explantation and reimplantation of the prosthesis concurrently with the reconstruction of the fragile disrupted annulus involving intimal entry using a patch or glue for a more detailed inspection. Despite the advancement in endovascular technology, an embolization in an endocardial entry tear might result in a catastrophic situation, such as aggravation to a full-thickness tear or rupture of the myocardium. Moreover, Tsukui et al. reported that the incidence of medical treatment was 28.1% among affected patients and the survival rate was 88.0% among patients who selected medical treatment [14]. In our case, conservative management with close observation was thought to be the best option, because the patient had a stable hemodynamic condition.

The time between the occurrence and disappearance of LAD in patients who were treated conservatively was considerably different among patients. In the present case, it was conceivable that the disappearance of the dissected cavity was rapid, because the pressurizing force from the left ventricle was applied between the left atrial myocardium and left atrial endocardium through the atrioventricular disrupted tissue; however, LAD was not persistent for a long time. Additionally, the small entry of the tear was occluded quickly and no communication between the dissected cavity and the atrial true lumen was noted.

We present a rare case of LAD following mitral valve replacement that was successfully treated with conservative management under close observation. The intraoperative routine use of TEE is an effective modality for promptly detecting the atrial dissection and ensuring accurate diagnosis. It is crucial to consider the hemodynamic conditions of the patient and the serial TEE follow-up examinations; when considering both, clinicians can decide if surgical or conservative treatment should be selected as the optimal treatment.

## Data Availability

The datasets supporting the conclusions of this article are included within the article.

## Abbreviations

LAD: left atrial dissection
TEE: Transesophageal echocardiography
